# The Facilitating Effect of Tartary Buckwheat Flavonoids and *Lactobacillus plantarum* on the Growth Performance, Nutrient Digestibility, Antioxidant Capacity, and Fecal Microbiota of Weaned Piglets

**DOI:** 10.3390/ani9110986

**Published:** 2019-11-18

**Authors:** Kai Cui, Qiang Wang, Shiqin Wang, Qiyu Diao, Naifeng Zhang

**Affiliations:** 1 Feed Research Institute, Chinese Academy of Agricultural Sciences, National Engineering Research Center of Biological Feed, Beijing 100193, China; cuikai@caas.cn (K.C.); fly87217@126.com (Q.W.); wshq1988@163.com (S.W.); diaoqiyu@caas.cn (Q.D.); 2 Institute of Animal Husbandry and Veterinary Science of Liangshan, Xichang 615042, China

**Keywords:** tartary buckwheat flavonoids, *Lactobacillus plantarum*, growth performance, antioxidant capacity

## Abstract

**Simple Summary:**

There has been a growing interest in the use of flavonoids and probiotics as alternatives of antibiotics in livestock production and as natural products for human health benefit. The effect of tartary buckwheat flavonoid supplementation in the diet has not been clearly investigated. The supplementation of tartary buckwheat flavonoids or *Lactobacillus plantarum* improved the growth performance, nutrient digestibility, and antioxidant capacity of weaned piglets. In combination, they exhibited a synergistic effect on nutrient digestibility.

**Abstract:**

Natural plant extracts and probiotics has been proved as the most preferred and effective alternatives for antibiotics in animal feeding. The current study aimed to investigate the facilitating effect of tartary buckwheat flavonoids and *Lactobacillus plantarum* on the growth performance, nutrient digestibility, antioxidant capacity, and microbiota of weaned piglets. Fifty 35-day-old weaned piglets (7.85 ± 0.67 kg) were randomly divided into five treatments with 10 piglets per treatment. Piglets in the negative control (NC) group were fed a basal diet, and others were fed basal diets supplemented with 40 mg/kg of colistin sulfate (positive control, PC), 10^9^ CFU/kg *Lactobacillus plantarum* (LP), 40 mg/kg of tartary buckwheat flavonoids (BF), and a combination of 10^9^ CFU/kg *Lactobacillus plantarum* and 40 mg/kg of tartary buckwheat flavonoids (LB). Supplementation of BF increased the average daily gain of piglets in the BF group (*p* < 0.05). The nutrient digestibility of piglets in the NC group was lower than that in other groups, while the digestibility of gross energy, dry matter, organic matter, and phosphorus of piglets in the LB group was higher than the other four groups (*p* < 0.05). Compared with the NC and pC group, supplementation of Lp increased the activity of superoxide dismutase (SOD), glutathione peroxidase (GSH-px), and catalase (CAT), while the BF increased the content of IgA and IgM (*p* < 0.05). Supplementation of colistin sulfate decreased the alpha diversity index, including chao and observed species, while the addition of Lp or combination of Lp and BF increased the abundance of *Selenomonas* or *Mitsuokella* in fecal samples, respectively. The results indicated that supplementation of *Lactobacillus plantarum* can improve the antioxidant capacity, while tartary buckwheat flavones can increase the growth performance and immune ability of weaned piglets. Moreover, in combination, they promote nutrient digestibility.

## 1. Introduction

Oxidative stress occurs when cellular reactive oxygen species (ROS) overwhelms the endogenous antioxidant defense capacity. Thus, redox homeostasis is not maintained, which is suggested to influence the development of the metabolic syndrome and neurodegenerative disorders [1]. At weaning, piglets are subjected to a number of stressors, which elevates pigs’ exposure to oxidative stress conditions [2]. Weaning is one of the most stressful events in a neonate’s life, which contributes to low feed intake, weight loss, diarrhea, intestinal dysfunction, and atrophy [3,4]. Antibiotics have its merits in increased body weight, decreased mortality and morbidity, and reduction in the occurrence of subclinical diseases. The subtherapeutic use of antibiotics, which has been used as a supplement to feed for a long period to solve postweaning problems, has turned into a global practice [5,6]. However, antibiotic residues in livestock products and the emergence of antibiotics resistant bacterial strains have remained a burning question in the world, and a number of countries have banned the use of subtherapeutic antibiotics in diet [7]. Therefore, alternatives to antibiotics for the alleviation and treatment of weaning stress are urgently needed. As the most preferred and effective alternatives for antibiotics in animal feeding, the beneficial effects of flavonoid-containing plant extracts and probiotics, namely antioxidative and intestinal health effects, have been shown in an increasing number of studies [8,9].

Flavonoids are part of the polyphenol class of phytonutrients and each type of flavonoid carries its own distinct set of actions and benefits. In recent years, a considerable number of studies focused on the effect of flavonoids, such as soy isoflavone, mulberry leaf flavonoids, or tengcha flavonoids, in improving the growth performance and health of livestock [10,11,12]. Tartary buckwheat, one of the major species of pseudocereals of the genus *Fagopyrum* in the *polygonaceae* family, is a popular nutritional food which has been reported to contain a great abundance of amino acids, vitamins, and flavonoids [13,14]. As plant-derived natural molecules, tartary buckwheat flavonoids have attracted research interest. Studies using the model animal exhibited a myriad of biological activities, such as antidiabetic [15,16], antioxidative, anti-hypertensive [17], and anti-inflammatory properties [14].

*Lactobacillus plantarum* (*L. plantarum*) is considered as probiotic which can compete with harmful gut flora colonization, maintain the gut integrity and stimulate the immune system of the host to increase the resistance to infectious agents [18,19]. Yang et al. (2014) reported that the supplementation of *L. plantarum* improved pig’s performance and effectively prevented diarrhoea by improving the function of the intestinal barrier in early life [20]. The cytological study showed that simultaneous application of *L. plantarum* and chlorogenic acid resulted in a protective effect against LpS-induced inflammation and oxidative stress in intestinal epithelial cells [21].

To the best of our knowledge, no study has investigated the application of tartary buckwheat flavonoids in the postweaning swine and the synergistic effect of flavonoids and probiotics has rarely been studied. The aim of the present study was therefore to examine the effect of dietary supplementation with tartary buckwheat flavonoids or *L. plantarum*, or their combinations on the growth performance, nutrient digestibility, antioxidant capacity and microbial diversity of weaned piglets.

## 2. Materials and Methods

This research was conducted at the Fang Shan pig breeding farm, Beijing, China (latitude 40.23′ N, longitude 116.60′ E). The Chinese Academy of Agricultural Sciences Animal Ethics Committee approved the experimental protocol, and all the methods conducted in this experiment were in accordance with humane animal care and handling procedures (AEC-CAAS-2017-02).

### 2.1. Animals, Diets and Management

The tartary buckwheat flavonoids selected in this research were purchased from MidWest bio-technology col., Ltd, Beijing, China. The crops for buckwheat flavonoids production were collected from the Sichuan province. According to the manufacturing company recommendations, the level of inclusion of tartary buckwheat flavonoids was 85% and the other 15% consisted of vitamin, microelement, and little rutin. The gradient addition test indicated the optimums doses for the growth performance was 40 mg/kg of diet. Fifty 35-day-old weaned piglets (Large White × Landrace) with an initial bodyweight of 7.85 ± 0.67 kg were randomly assigned to five treatments with 10 piglets per treatment, and the piglets were housed individually. The treatments were as follows: (1) Negative control (NC): Piglets were fed with basal diets; (2) positive control (pC): Piglets were fed with basal diets supplemented with 40 mg/kg of colistin sulfate (Zhongnongxing Feed SCI. & Tech. Co., Ltd, Beijing, China); (3) *L. plantarum* (Lp): Piglets were fed with basal diets supplemented with 10^9^ CFU/kg of *L. plantarum* (JN560899.1); (4) tartary buckwheat flavonoids (BF): Piglets were fed with basal diets supplemented with 40 mg/kg of tartary buckwheat flavonoids; and (5) *L. plantarum* and tartary buckwheat flavonoids (LB): Piglets were fed basal diets supplemented with 10^9^ CFU/kg of *L. plantarum* and 40 mg/kg of tartary buckwheat flavonoids. The basal diet was formulated following NRC (2012) recommendations to meet the nutrient requirements of weaned piglets and free of antibiotics or other additives (Table 1). The feed and water were available *ad libitum* during the experimental period. Feed offered and refusals and body weight were recorded at the beginning and end of the experiment to calculate the feed consumption, average daily gain (ADG), average daily feed intake (ADFI), and feed efficiency (F:G ratio). The trial lasted 28 days.

### 2.2. Digestibility Trial and Chemical Analyses

Piglets were fed diets mixed with 0.1% titanium dioxide (TiO_2_) as exogenous indigestible marker to determine apparent digestibility of nutrients during the last 10 days of the trial. After four days of adaptation, freshly voided feces were collected by grab sampling from the pen floors of six randomly selected piglets of each treatment for six further days. All diets and fecal samples were stored immediately at −20 °C until analysis. Feces were then thawed, homogenized, subsampled, dried in an air-forced oven (Model FC-610, Advantec, Toyo Seisakusho Co. Ltd., Tokyo, Japan) at 65 °C for 48 h, and ground into 1-mm particles in a centrifugal mill (model ZM200; Retsch GmbH, Haan, Germany) for chemical analyses.

The feces and diets were analyzed for dry matter (DM, method 930.15; AOAC 1990), crude protein (Cp, 6.25 × N; method 984.13; AOAC 1990), and gross energy (GE) using an automatic adiabatic oxygen bomb calorimeter (C200; IKA Works Inc., Staufen, Germany). The contents of ash (method 942.05), ether extract (EE; method 920.39), phosphorus (p, method 965.17), and calcium (Ca, method 968.08) of the feces and diets were conducted according to the methods of AOAC (1990). The TiO_2_ content was measured by the method of Myers et al. (2004) [22]. The apparent digestibility of GE, DM, OM, Cp, EE, Ca, and p were calculated using the marker concentration of feces relative to feed by the indicator method [23].

### 2.3. Blood Profiles

By the end of the trail, blood samples were collected via anterior vena cava puncture into vacuum tubes from the same piglets, which provided the fecal samples for measuring the concentration of SOD, GSH-px, CAT, and the concentration of malondialdehyde (MDA) after piglets fasted for 3 h. Blood samples were centrifuged for 15 min at 3400 rcf and the serums were removed to 1.5-mL plastic tubes and stored at −20 °C. The activity of SOD, GSH-px, and CAT and the concentration of MDA were analyzed with commercial kits (Nanjing Jiancheng Bioengineering Institute, Jiangsu, China) according to the manufacturer’s protocols. Blood urea nitrogen (BUN), glucose, and immunoglobulin were analyzed by a biochemical auto analyzer (Hitachi automatic biochemical analyzer 7600, Tokyo, Japan) using commercial kits.

### 2.4. DNA Extraction, pCR Amplification of 16S rRNA and Illumina Hiseq Sequencing

Fecal samples were collected from each pen via rectal massage and microbial DNA was extracted using a commercially available kits (Omega Bio-tek, Norcross, GA, USA) according to the manufacturer’s protocols. The universal primers 341F (5′-barcode-CCTAYGGGRBGCASCAG-3′) and 806R (5′-GGACTACCVGGGTATCTAAT-3′) were used to obtain the pCR amplicons for paired-end sequencing on an Illumina HiSeq platform. The amplification and 16S rRNA gene high-throughput sequencing were performed in Realbio Genomics Institute (Shanghai, China). 

### 2.5. Processing of Sequencing Data

The raw Illumina fastq files were subjected to a quality control procedure using UpARSE. After quality control and filtering chimeras, the assembled sequences were clustered to generate operational taxonomic units (OTUs) at a 97% identity threshold using USEARCH. The representative sequence of each OTU was assigned to a taxonomic level in the RDp database using the RDp classifier with a confidence threshold of 80%. The α diversity index, including Chao1, Shannon, and Simpson were calculated with Mothur. The sequencing data obtained in this study were deposited in the NCBI Sequence Read Archive (SRA) under accession numbers SRR6334389 to SRR6334415.

### 2.6. Statistical Analysis

The relative abundances of communities do not fit normal distribution, and arcsine transformation function was performed before analyses. All data were analyzed by one-way ANOVA using SAS (version 9.1, SAS Institute, Inc., Cary, NC, USA; 2004). Statistical differences among the means of the treatments were compared using the Duncan’s Multiple Range Test. Treatment differences with *p* < 0.05 were considered statistically significant and 0.05 ≤ *p* < 0.10 were designated as a tendency.

## 3. Results

### 3.1. Growth Performance of Piglets

The initial bodyweight showed no difference among the four treatments (*p* > 0.05). The final body weight (FBW) and ADG of piglets in the NC, pC, Lp and LB group showed no significant difference, while the ADG of piglets in the BF group was higher than that in the other four groups (*p* < 0.05, Table 2). Compared with the NC and pC group, piglets of the BF group showed an increase in feed efficiency, but not significantly different from the other four groups (*p* > 0.05).

### 3.2. Nutrient Digestibility Analysis

The digestibility of GE, DM, OM, Cp, EE, Ca, and p of piglets in the pC, Lp, BF, and LB group was higher than that in NC group (*p* < 0.05). The digestibility of GE, DM, OM, and p of piglets in the LB group was higher than that in other groups (*p* < 0.05, Table 3).

### 3.3. Blood Biochemical Parameters

The concentration of the blood urea nitrogen of piglets in pC and BF group was higher than that in the other three groups (*p* < 0.05). The supplementation of tartary buckwheat flavonoids and compound of tartary buckwheat flavonoids and *L. plantarum* increased the concentration of the blood glucose of piglets (*p* < 0.05). Compared to the NC and Lp group, supplementation of tartary buckwheat flavonoids increased the concentration of IgA, but no significant difference was found among the other four groups (*p* < 0.05). The concentration of IgM of piglets in the BF and LB group was higher than that in other three groups (*p* < 0.05, Table 4).

### 3.4. Antioxidant Enzyme Activities

**Figure 1:**
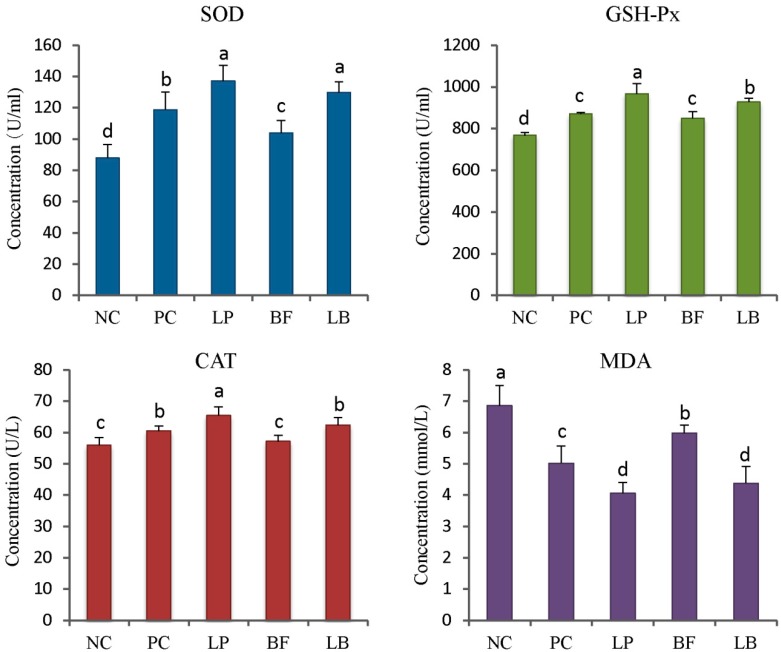
Effects of *L. plantarum*, tartary buckwheat flavones, and their combination on the activities of antioxidant enzymes of weaned piglets. SOD: Superoxide dismutase; GSH-px: Glutathione peroxidase; CAT: Catalase; MDA: Malondialdehyde.

Piglets from the Lp and LB group had higher SOD activity than that of the NC, pC, and BF group (*p* < 0.05). The activity of GSH-px and CAT of Lp group were higher than that of other four groups (*p* < 0.05). The GSH-px activity of LB group was higher when compared with the NC, pC, and BF group (*p* < 0.05). Compared with the NC, pC, and BF group, the MDA content of weaned piglets from the Lp and LB group was decreased (*p* < 0.05, Figure 1).

### 3.5. Sequencing Depth and Index of Microbial Community

Illumina HiSeq sequencing generated a total of 1,431,031 clean reads with an average of 53,001 ± 10,687 reads per sample after data filtering, quality control, and removal of primers, chimeras, and low-confidence singletons. All reads were classified into 538 operational taxonomic units (OTUs) based on 97% nucleotide sequence identity between reads.

**Figure 2:**
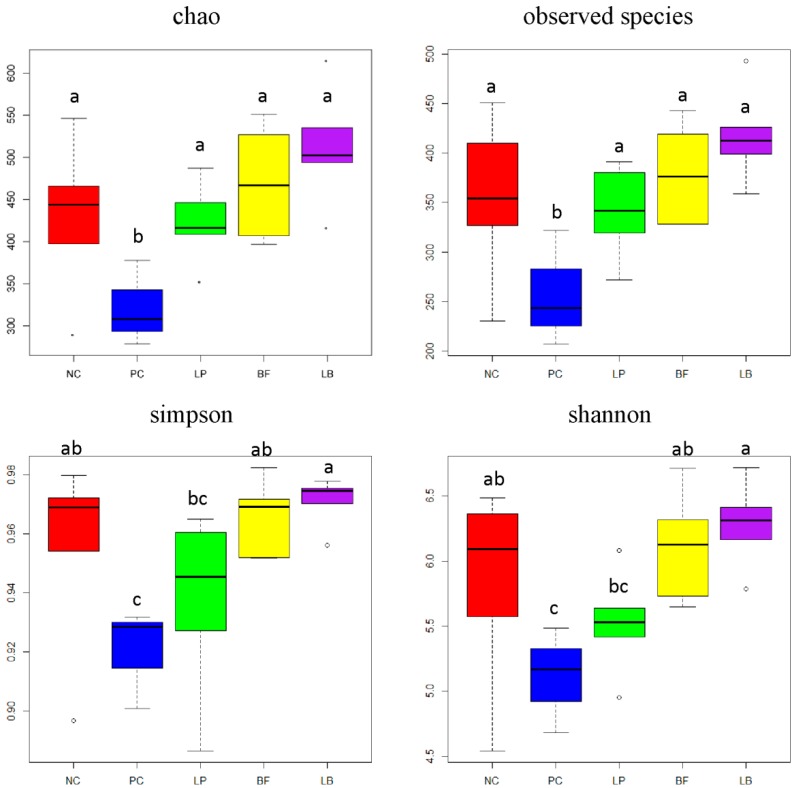
Effects of *L. plantarum*, tartary buckwheat flavones, and their combination on the community richness estimates and diversity indices of weaned piglets.

The saturation plateau of rarefaction curves and species accumulation curves indicated that sampling had sufficient coverage and depth to accurately describe the bacterial composition of each group (Figure S1). The OTU number of pC, NC, Lp, BF, and LB was 264, 361, 348, 386, and 424, respectively. The pC and NC groups had lower OTU numbers compared with other groups (*p* < 0.05). The community diversity index of Chao and Observed species was different (*p* < 0.05) between the pC group and other groups in weaned piglets (Figure 2). The Simpson and Shannon index showed no difference between the pC and Lp group, but differed significantly with other three groups.

### 3.6. Relative Abundance and Diversity of Bacterial Communities

**Figure 3:**
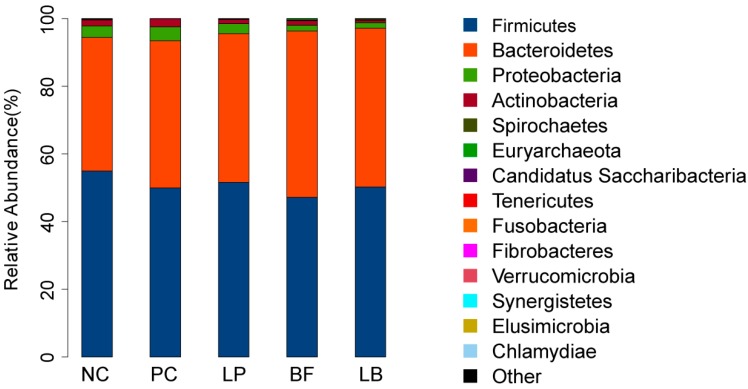
Effects of *L. plantarum*, tartary buckwheat flavones and their combination compared to the dominant bacterial phyla. The color-coded bar plot shows the relative abundances of different genera across different groups.

At the phylum level, 15 phyla were identified in the samples from the fecal samples of weaned piglets (Figure 3 and Table 5). Five groups showed similar taxonomic compositions at the phylum level, which were dominated by *Firmicutes, Bacteroidetes, Proteobacteria*, and *Actinobacteria*. However, the relative abundance of those phyla varied considerably among the five groups (Table 4). The *Firmicutes* and *Bacteroidetes* dominated in all the treatment groups. The phylum *Proteobacteria* and *Actinobacteria* were more prominent in samples taken from the BF and LB groups when compared with the Lp, pC, and NC groups.

**Figure 4:**
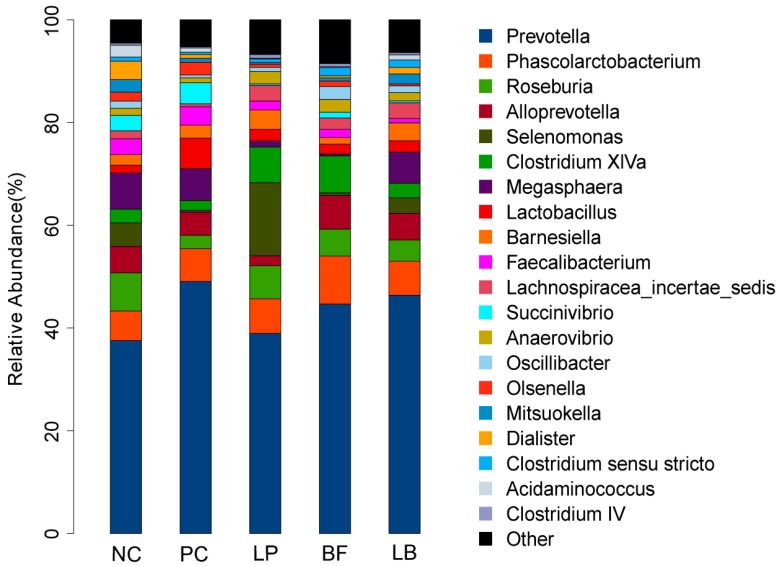
Effects of *L. plantarum*, tartary buckwheat flavones, and their combination on comparison of the dominant bacterial genera o. The color-coded bar plot shows the relative abundances of different genera across different groups.

At the genus level, 114 genera belonging to the 15 phyla were detected in the samples. In general, the 19 most abundant genera (the relative abundance of genera representing more than 1% of the five libraries) were present across different groups, and the relative abundance levels were markedly different among the different treatment groups (Figure 4 and Table 6). The *Prevotella* and *Selenomonas* made up the main bacterial species in the Lp group, with *Selenomonas* having the highest relative abundances among the five groups. In the LB group, the *Prevotella* was predominant with an abundance of 46.34%, followed by *Phascolarctobacterium*, *Megasphaera*, and *Roseburia*. The *Mitsuokella* of the LB group was the most abundant taxa compared with the other four groups.

## 4. Discussion

In the current study, the supplementation of tartary buckwheat flavonoids significantly increased the ADG, which is in accordance with the reports of Zhao et al. [24] that dietary tartary buckwheat flavonoids increased the bodyweight and ADG of lamb. Similarly, flavonoids from alfalfa and *Allium mongolicum Regel* increased the growth performance of growing rabbits, geese, and sheep, respectively [25,26]. Furthermore, previous studies reported that the similar structure of flavonoids to estradiol, which regulates the secretion of growth hormone, can accelerate protein synthesis or stimulate IGF-1 to promote the body weight gain [27,28,29,30]. However, previous studies showed the inconsistent result of the effect of probiotics on growth performance. Le et al. (2016) reported that feeding fermented wheat with *L. reuteri* did not affect the growth performance of weaned piglets, while Cheng reported that the daily weight gain of weaned piglets was improved by a diet supplemented with *L. plantarum* [31]. In this research, supplementation with *L. plantarum* had no significant effect on the growth performance of weaning piglets. The composition of diets, strains, and addition amount might explain the contradictory results on the use of *L. plantarum* in weaned piglets.

Previous studies have confirmed that enhanced digestibility of nutrients could promote the absorption and improve the growth performance of livestock. In the present study, the supplementation of colistin sulfate, *L. plantarum*, and tartary buckwheat flavonoids increased the digestibility of GE, DM, OM, Cp, EE, Ca, and p, which is in accordance with the study of Meng et al. (2010), who reported that supplementation of *Clostridium butyricum endospore complex* had beneficial effects on apparent total tract digestibility of finishing piglets [32]. Studies in weaning piglets indicated that dietary supplementation with probiotics improved the growth performance and nutrition digestibility [33]. The promoting digestion function of probiotics might attribute to the increased secretion and activity of digestive enzymes [34]. Compared with *L. plantarum* or tartary buckwheat flavonoids fed to piglets alone, dietary supplementation of the combination of *L. plantarum* and tartary buckwheat flavonoids significantly improved the digestibility of GE, DM. and p. Previous studies have verified the combined beneficial effects of probiotics in broilers [35], weaning rabbits [36], and growing piglets [37]. However, further research is needed to investigate the possible mechanism of combined effects.

Serum parameters represent an integrated index of nutrient supply in relation to the utilization of nutrients. BUN is a marker of the protein metabolism and dietary amino acid balance which reflects the N utilization in the animal [38]. In the current study, the increased concentration of BUN indicated that supplementation of tartary buckwheat flavonoids increased the protein utilization, which might be the possible cause of improved growth performance of the piglets. This effect warrants future research. Extensive studies have provided a wealth of information on the modulation of immune ability of flavonoids [39]. In this research, the supplementation of tartary buckwheat flavonoids increased the concentration of IgA, IgG, and IgM of the weaned piglets, which is in accordance with a number of in vitro studies reporting the role of flavonoids in the regulation of immune response [40]. The role of flavonoid in the modulation of immune system is substantiated by the data from this research.

Oxidative stress is recognized as an imbalance between reactive oxygen species (ROS) level and antioxidant mechanism activity and is considered one of the primary determinants of aging and carcass quality [41]. Increasing experimental evidence indicated that probiotics and flavonoids exert beneficial antioxidant effects [42]. In the present study, dietary supplementation of *L. plantarum* significantly increased the activities of SOD, GSH-px, and CAT, which is inconsistent with previous observations that *L. plantarum* markedly elevated the gene expression of several antioxidant genes such as GR, GSH-px, and SOD by Nrf2-mediated signal pathway [43,44]. Suzuki et al. (2013) identified two compounds that demonstrated 2, 2-diphenyl-1-picrylhydrazy (DppH) radical scavenging activity from cultures of *L. plantarum*, which might be the underlying antioxidant mechanism of *L. plantarum* [41]. Compared with the control group, the supplementation of tartary buckwheat flavonoids improved the activity of SOD and GSH-px in this research. However, the activity of antioxidant enzymes was significantly lower than that of the *L. plantarum* and combination treatments. The antioxidant activity can be attributed to the various bioactive compounds present in buckwheat, such as the total flavonoid content, which was positively correlated to the antioxidant activity of the tartary buckwheat [45]. Supplementation with buckwheat honey also resulted in strong DppH radical scavenging activity and potential ferric reducing antioxidant activity, and enhanced hepatic antioxidant enzymes such as SOD and GSH-px [41]. The discrepancy might attribute to the level of inclusion of tartary buckwheat flavonoids, which needs further research.

Previous studies revealed that correlation of antioxidant status and gut microbiota of the mouse and HT-29 cell model were supplemented with *L. plantarum*, and studies of flavonoid metabolism indicated that intestinal bacteria are deeply involved in the interaction between metabolites and intestinal microbiota [35,42,46]. In the present study, HiSeq sequencing of 16S rRNA was used to evaluate the changes in the fecal bacteria community of weaned piglets. The diversity indices and observed species were increased in the LB group compared with the other groups, suggesting that the combination of *L. plantarum* and tartary buckwheat flavonoids increased the intestinal community diversity in weaned piglets and a synergistic effect of *L. plantarum* and tartary buckwheat flavonoids on microbial diversity existed. In the current study, the community richness index of Chao and Observed species significantly decreased in the pC group compared with all other groups, suggesting that the addition of colistin sulfate decreased the intestinal microbial richness in weaned piglets, and the antimicrobial activity of antibiotics might be one of the reasons [47].

We found that *Firmicutes, Bacteroidetes*, and *Proteobacteria* were the dominant phyla among the groups, which was consistent with the studies of Ley et al. [48] and de Oliveira et al. [49], who reported that *Firmicutes* and *Bacteroidetes* were the most numerically dominant phyla in the microbiome of terrestrial mammals. The treatments shared 114 genera, and the 19 most abundant genera (the relative abundance of genera representing more than 1% of the five libraries) were present in all samples across the different treatments. The relative abundance of the genera from this shared community varied considerably among the groups. *Megasphaera* sp. is a normal inhabitant in the rumen of cattle and sheep and is also found in the feces and intestine of humans and piglets [50]. In the current study, the genus *Megasphaera* was significantly more abundant in the pC group than in the Lp and BF groups. Shetty et al. reported that *Megasphaera* has several mechanisms for protection against oxidative stress and harbor multidrug resistance efflux pumps and genes that confer resistance to specific antibiotics, which might account for the growth of *Megasphaera* [51]. *Selenomonas* species are colonizers of the digestive system where they act at the interface between health and disease [52]. In the current study, the genus *Selenomonas* was significantly more abundant in the Lp group compared with the other four groups. Owing to their fastidious and incomplete known growth requirements, a large number of *Selenomonas* was not cultured and the mechanism of more abundant in *L. plantarum* treatment needs further research.

## 5. Conclusions

This study demonstrated that the supplementation of tartary buckwheat flavonoids increased the average daily gain and immune ability, while the supplementation of *L. plantarum* improved the antioxidant capacity of weaned piglets. The combination of tartary buckwheat flavonoids and *L. plantarum* increased the nutrient digestibility of piglets and their combination had a synergistic effect. Compared to the antibiotic addition group, the supplementation of tartary buckwheat flavonoids and *L. plantarum* increased the community diversity, while the addition of *L. plantarum* or their combination increased the abundance of *Selenomonas* or *Mitsuokella*, respectively. The results provided the new information regarding the facilitating effect of tartary buckwheat flavonoids, *L. plantarum*, and their compound on weaned piglets and could be the alternatives of colistin sulfate.

## Figures and Tables

**Table 1:** Composition and nutrient levels of the basal diet (dry matter basis).

Items	Content
Ingredients	
Corn	59
Soybean meal	16.5
Wheat bran	5
Rice polishings	5
DDGS	5
Wheat middling	5
Fat powder	0.5
premix ^a^	4
Nutrient levels ^b^	
DM	88.03
Cp	18.95
DE (MJ/kg)	15.43
EE	2.90
Ash	5.41
phosphorus	0.58
Calcium	0.68
Lys	1.11
Met	0.32
Cys	0.36
Thr	0.70

^a^ The premix provided the following nutrients per kilogram of the diet: VA 8000 IU, VD_3_ 3000 IU, VE 20 IU, VK_3_ 2 mg, VB_1_ 4 mg, VB_2_ 3.6 mg, VB_5_ 40 mg, VB_6_ 4 mg, VB_12_ 0.02 mg, biotin 0.15 mg, folic acid 1.0 mg, *D*-pantothenic acid 11 mg, nicotinic acid 10 mg, antioxidant 100 mg, Cu 10 mg, Fe 80 mg, Mn 80 mg, Zn 75 mg, I 0.40 mg, Se 0.30 mg. ^b^ DE was a calculated value and others were measured values. DM: Dry matter; Cp: Crude protein; DE: Digestible energy; EE: Ether extract.

**Table 2:** Effects of *L. plantarum*, tartary buckwheat flavones, and their compound on growth performance of weaned piglets.

Items ^1^	Treatments ^2^					SEM	*p*-Value
	NC	pC	Lp	BF	LB		
IBW, kg	7.81	7.84	7.87	7.76	7.85	0.67	0.998
FBW, kg	13.70 ^b^	14.55 ^ab^	14.37 ^b^	16.76 ^a^	15.90 ^ab^	0.36	0.047
ADFI, g	461.12	554.04	466.11	525.04	537.64	-	-
ADG, g	210.39 ^b^	239.56 ^b^	232.14 ^b^	321.34 ^a^	287.43 ^ab^	12.32	0.029
F/G	2.19 ^ab^	2.31 ^a^	2.01 ^abc^	1.63 ^c^	1.87 ^bc^	0.11	0.082

^1^ IBW: Initial body weight; FBW: Final body weight; ADFI: Average daily feed intake; ADG: Average daily gain. Letters shared in common between the groups indicate no significant difference (*p* > 0.05). ^2^ NC: Negative control; pC: Positive control; Lp: *L. plantarum*; BF: Tartary buckwheat flavonoids; LB: *L. plantarum* and tartary buckwheat flavonoids.

**Table 3:** Effects of *L. plantarum*, tartary buckwheat flavones, and their compound on nutrient digestibility of weaned piglets.

Items ^1^	Treatments ^2^					SEM	*p*-Value
	NC	pC	Lp	BF	LB		
DM (%)	82.19 ^c^	84.03 ^b^	84.31 ^b^	84.26 ^b^	86.28 ^a^	0.264	<0.001
OM (%)	85.25 ^c^	87.07 ^b^	87.21 ^b^	87.19 ^b^	88.70 ^a^	0.227	<0.001
GE (%)	81.11 ^c^	83.34 ^b^	83.48 ^b^	83.88 ^b^	85.19 ^a^	0.277	<0.001
Cp (%)	76.50 ^b^	80.50 ^a^	80.79 ^a^	81.69 ^a^	81.68 ^a^	0.423	<0.001
EE (%)	33.32 ^b^	58.37 ^a^	61.24 ^a^	60.45 ^a^	62.59 ^a^	2.242	<0.001
Ca (%)	35.55 ^c^	41.92 ^b^	42.88 ^b^	44.44 ^ab^	49.46 ^a^	1.132	<0.001
p (%)	50.36 ^c^	54.92 ^b^	55.48 ^b^	55.27 ^b^	59.95 ^a^	0.752	<0.001

^1^ DM: Dry matter; OM: Organic matter; GE: Gross energy; Cp: Crude protein; EE: Ether extract; Ca: Calcium; p: Phosphorus. Letters shared in common between the groups indicate no significant difference (*p* > 0.05). ^2^ NC: Negative control; pC: Positive control; Lp: *L. plantarum*; BF: Tartary buckwheat flavonoids; LB: *L. plantarum* and tartary buckwheat flavonoids.

**Table 4:** Effects of *L. plantarum*, tartary buckwheat flavones, and their compound on serum biochemical and immune indices of weaned piglets.

Items ^1^	Treatments ^2^					SEM	*p*-Value
	NC	pC	Lp	BF	LB		
BUN, mmol/L	6.68 ^c^	7.87 ^ab^	7.17 ^bc^	8.48 ^a^	7.49 ^bc^	0.17	0.003
GLU, mmol/L	2.54 ^c^	3.67 ^ab^	2.84 ^bc^	4.17 ^a^	3.94 ^a^	0.17	0.003
IgG, g/L	7.54 ^c^	8.29 ^ab^	7.65 ^bc^	8.78 ^a^	8.15 ^abc^	0.13	0.005
IgA, g/L	0.89 ^b^	0.86 ^b^	0.85 ^b^	1.16 ^a^	0.99 ^b^	0.03	0.004
IgM, g/L	0.69 ^c^	0.75 ^bc^	0.71 ^bc^	0.87 ^a^	0.80 ^ab^	0.02	0.003

^1^ BUN: Blood urea nitrogen; GLU: Glucose; IgG: Immunoglobulin G; IgA: Immunoglobulin A; IgM: Immunoglobulin M. ^2^ NC: Negative control; pC: Positive control; Lp: *L. plantarum*; BF: Tartary buckwheat flavonoids; LB: *L. plantarum* and tartary buckwheat flavonoids. Letters shared in common between the groups indicate no significant difference (*p* > 0.05).

**Table 5:** Effects of *L. plantarum*, tartary buckwheat flavones, and their combination on comparison of the dominant genus of microbes.

Items	Treatments ^a^					SEM	*p*-Value
	NC	pC	Lp	BF	LB		
*Firmicutes*	47.21	50.25	51.62	54.93	49.96	4.51	0.7636
*Bacteroidetes*	49.10	46.86	43.90	39.49	43.44	4.35	0.519
*Proteobacteria*	1.70	1.68	2.97	3.42	4.13	1.04	0.4251
*Actinobacteria*	1.37	0.63	1.23	1.76	2.41	0.53	0.2762

^a^ NC: Negative control; pC: Positive control; Lp: *L. plantarum*; BF: Tartary buckwheat flavonoids; LB: *L. plantarum* and tartary buckwheat flavonoids.

**Table 6:** Effects of *L. plantarum*, tartary buckwheat flavones, and their combination on comparison of the dominant genus of microbes.

Phylum	Genus	Relative Abundance (%) ^1^					SEM	*p*-Value
		NC	pC	Lp	BF	LB		
*Firmicutes*	*Phascolarctobacterium*	6.54	6.35	7.14	9.38	6.61	1.07	0.2422
	*Lactobacillus*	1.32	5.93	0.94	1.91	2.20	1.23	0.1686
	*Clostridium sensu stricto*	0.94	0.44	0.71	1.59	1.46	0.52	0.5263
	*Clostridium XlVa*	3.12	1.88	7.19	7.17	2.85	1.70	0.1103
	*Lachnospiracea_incertae_sedis*	1.74	0.57	3.29	2.22	3.07	0.58	0.0514
	*Roseburia*	8.02	2.62	7.42	5.20	4.15	1.96	0.3769
	*Faecalibacterium*	1.60	3.58	1.87	1.58	0.88	0.73	0.2635
	*Oscillibacter*	1.63	0.57	0.94	2.49	1.34	0.61	0.2664
	*Acidaminococcus*	1.37	0.82	0.05	0.15	0.97	0.39	0.1065
	*Anaerovibrio*	1.64	0.94	2.85	2.53	1.57	0.91	0.6526
	*Dialister*	2.04	0.72	0.02	0.85	1.29	0.70	0.574
	*Megasphaera*	2.62 ^ab^	6.23 ^a^	0.08 ^b^	0.40 ^b^	6.08 ^a^	1.38	0.0094
	*Mitsuokella*	0.83 ^a^	0.79 ^a^	0.30 ^a^	0.63 ^a^	1.94 ^b^	0.36	0.0286
	*Selenomonas*	4.02 ^b^	0.30 ^b^	11.2 ^a^	0.47 ^b^	2.35 ^b^	1.70	0.0019
*Bacteroidetes*	*Prevotella*	43.22	43.56	43.56	44.62	46.34	3.66	0.8588
	*Barnesiella*	1.86	2.54	1.20	1.27	3.48	1.12	0.5283
	*Alloprevotella*	6.08	4.51	1.70	6.57	5.17	1.28	0.0944
*Proteobacteria*	*Succinivibrio*	3.56	4.12	0.31	1.11	0.39	1.18	0.1252
*Actinobacteria*	*Olsenella*	1.53	2.48	0.63	1.05	0.37	0.60	0.2371

^1^ NC: Negative control; pC: Positive control; Lp: *L. plantarum*; BF: Tartary buckwheat flavonoids; LB: *L. plantarum* and tartary buckwheat flavonoids. In the same row, values with no letter or the same letter superscripts indicate no significant difference (*p* > 0.05), while with different small letter superscripts indicate significant difference (*p* < 0.05).

## Supplementary Materials

The following are available online at https://www.mdpi.com/2076-2615/9/11/986/s1.
