# Supplementary figures and images for: The Facilitating Effect of Tartary Buckwheat Flavonoids and Lactobacillus plantarum on the Growth Performance, Nutrient Digestibility, Antioxidant Capacity, and Fecal Microbiota of Weaned Piglets
Source: Animals (Basel). 2019 Nov 18;9(11):986. doi: 10.3390/ani9110986 (PMC6912274; doi:10.3390/ani9110986)

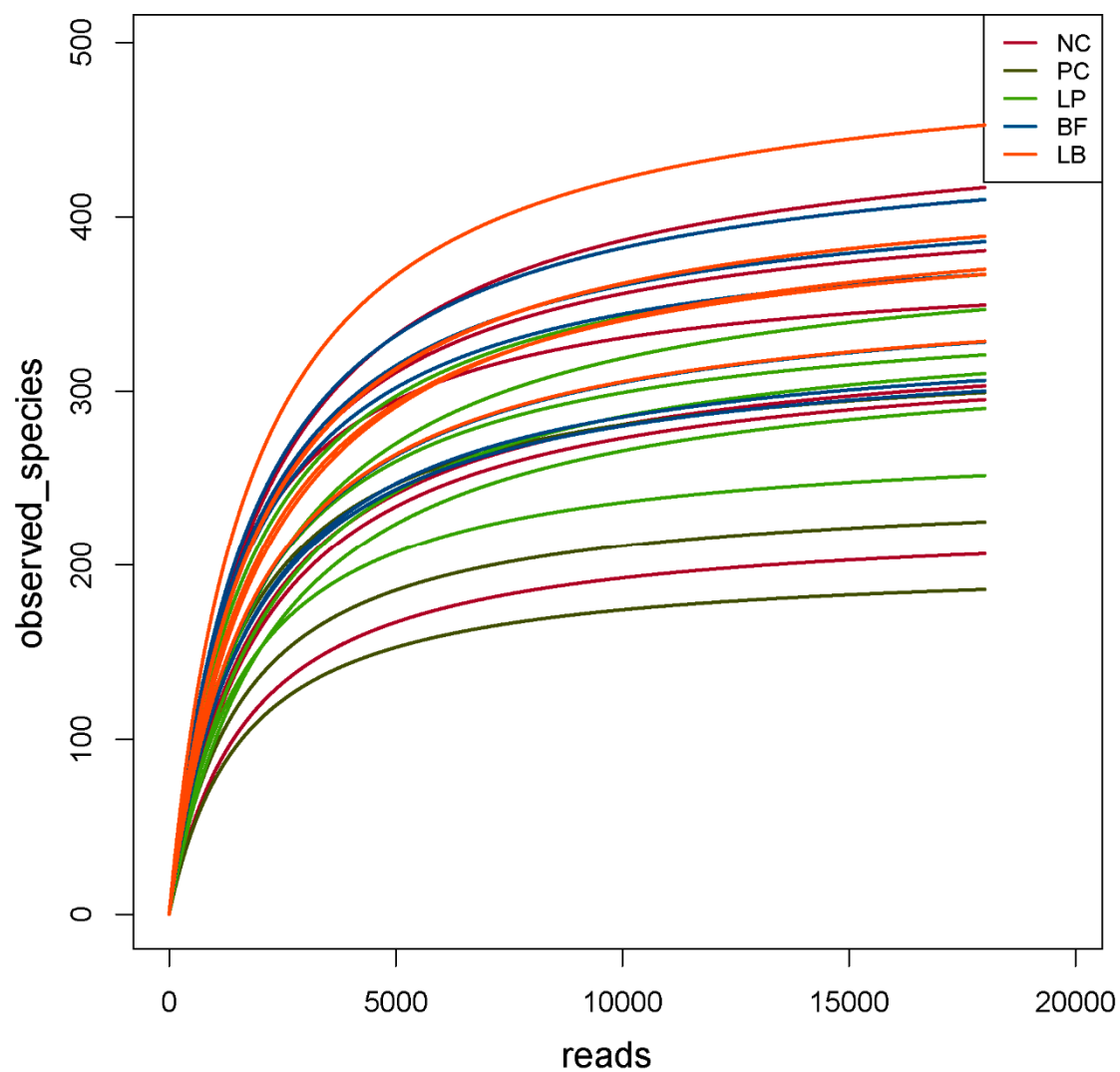

**Figure S1.** Rarefaction analysis of the different samples.

Supplement: Supplementary file 1 [file animals-09-00986-s001.pdf]
